# Ensartinib for EML4-ALK-positive lung adenocarcinoma with comorbid mutations in TP53, EGFR, and ERBB2: a case report

**DOI:** 10.3389/fonc.2025.1520287

**Published:** 2025-02-14

**Authors:** Xiaoqing Huang, Lingxian Zhou, Jianyong Xia, Haifeng Jian, Jinji Liu, Yunying Huang, Qingsheng Chen

**Affiliations:** Department of Oncology and Hematology, The Second People's Hospital of Foshan, Foshan, China

**Keywords:** advanced lung adenocarcinoma, ensartinib, EML4-ALK+, multiple gene fusion, case report

## Abstract

**Background:**

In non-small cell lung cancer (NSCLC), anaplastic lymphoma kinase (ALK) gene rearrangements are commonly detected in lung adenocarcinoma. ALK-positive (ALK+) patients may occasionally exhibit concurrent genetic alterations that potentially impact prognosis. New therapeutic strategies are needed for ALK+ NSCLC patients with multiple simultaneous gene mutations.

**Case presentation:**

A 58-year-old man was diagnosed with lung adenocarcinoma (stage IVB, T4N3M1c) with an echinoderm microtubule-associated protein-like 4-ALK+ (EML4-ALK+) rearrangement, harboring tumor protein 53 (TP53), epidermal growth factor receptor (EGFR), and receptor tyrosine-protein kinase erbB-2 (ERBB2) mutations. After three cycles of chemotherapy, the patient developed intolerance. Subsequently, ensartinib (225 mg daily) was administered orally on April 14, 2021. After 3 months of ensartinib treatment, the patient achieved a partial response and reached stable disease at six months, which sustained for 30 months till April 8, 2024, with grade 1 rash and no brain metastases. Currently, the patient remains on ensartinib treatment, without disease progression.

**Conclusion:**

This case demonstrates the potential for ensartinib in the treatment of EML4-ALK+ lung adenocarcinoma with multiple gene mutations. Further investigation through clinical trials is needed to evaluate the safety and efficacy of this targeted therapy.

## 1 Introduction

Non-small cell lung cancer (NSCLC), particularly lung adenocarcinoma, is predominantly driven by mutations in driver genes, primarily including anaplastic lymphoma kinase (ALK) rearrangements and epidermal growth factor receptor (EGFR) mutations (1). ALK rearrangements occur in approximately 3–10% of NSCLC patients, and echinoderm microtubule-associated protein-like 4 (EML4) is the most common ALK fusion partner in lung adenocarcinoma (2, 3). EGFR activation mutations are another major targetable oncogenic mutation in advanced NSCLC, found in 20–50% of NSCLC patients (4, 5). In recent years, targeted therapy has revolutionized the treatment landscape of NSCLC. ALK-tyrosine kinase inhibitors (ALK-TKIs) and EGFR-TKIs are recommended as the standard of care for NSCLC patients with ALK rearrangements or EGFR mutations, respectively (6). Nevertheless, these treatment approaches are typically limited to patients with single-gene mutations, and current therapeutic options for conditions involving multiple genes remain considerably limited.

ALK rearrangements and EGFR mutations are typically considered mutually exclusive (2). Although the proportion of concurrent EGFR/ALK co-mutation in NSCLC was rare, some cases or studies reported the occurrence of this situation (7–12), underscoring the necessity for accurate biomarker testing to identify subgroups of NSCLC with oncogenic drivers before treatment. ESMO Clinical Practice Guideline of oncogene-addicted metastatic non-small-cell lung cancer also recommends testing for driver genes in adenocarcinoma patients prior to treatment to guide treatment decisions (1). While several studies have reported on the treatment of patients with the coexistence of EGFR and ALK rearrangement, there is no reported case of a patient with EML4-ALK-positive (EML4-ALK+) lung adenocarcinoma, harboring concurrent mutations in tumor protein 53 (TP53), EGFR, and receptor tyrosine-protein kinase erbB-2 (ERBB2). New strategies need to be explored for those with multiple gene fusions.

Herein, we presented a patient with EML4-ALK+ lung adenocarcinoma with multiple metastases harboring multiple gene mutations, including TP53, EGFR, and ERBB2. The patient achieved a partial response (PR) after 3 months of ensartinib treatment and reached stable disease (SD) at six months, which was sustained for 30 months. This case deepens our understanding of ALK+ NSCLC with diverse genomic alterations and provides insight into the new treatment of EML4-ALK+ lung adenocarcinoma with multiple gene fusions.

## 2 Case presentation

A 58-year-old man with no history of tobacco, alcohol, or dust exposure was admitted for choking sensation during swallowing persisting for over six months on January 3, 2021, with a performance status (PS) score of 1. His medical history included hypertension and a gastrectomy for gastric bleeding. He has a family history of primary hepatocellular carcinoma. Physical examination revealed a firm, fixed, enlarged left supraclavicular lymph node with a maximum diameter of approximately 2 cm.

**Figure 1:**
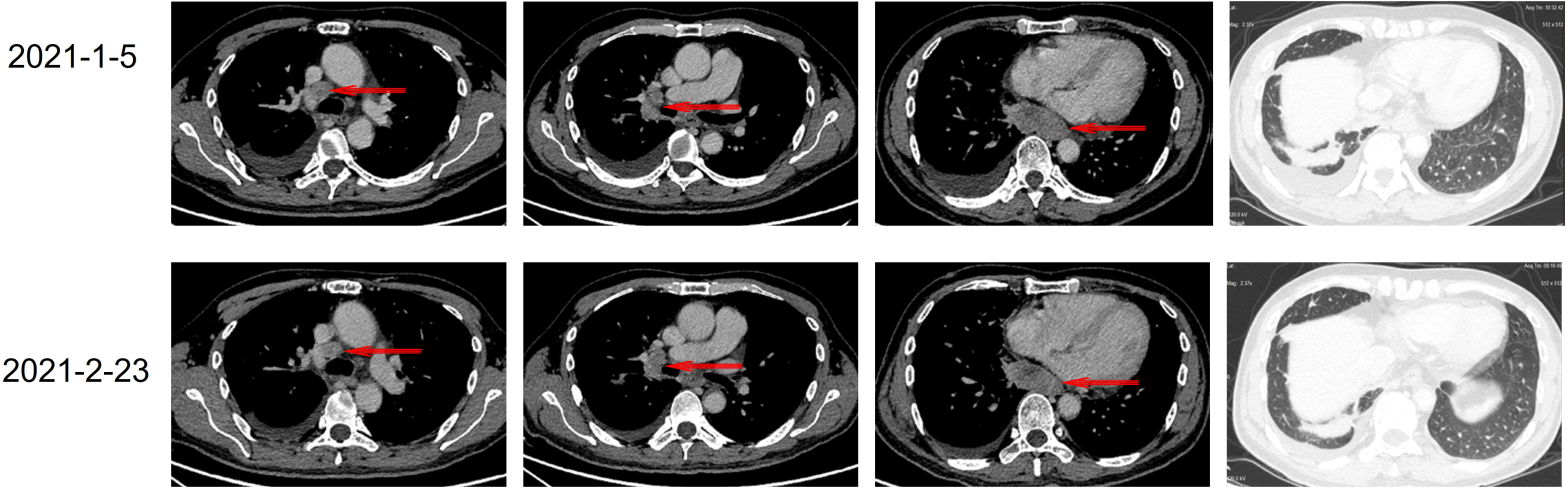
Radiological images of the patient before treatment and after 1 cycle of chemotherapy.

**Figure 2:**
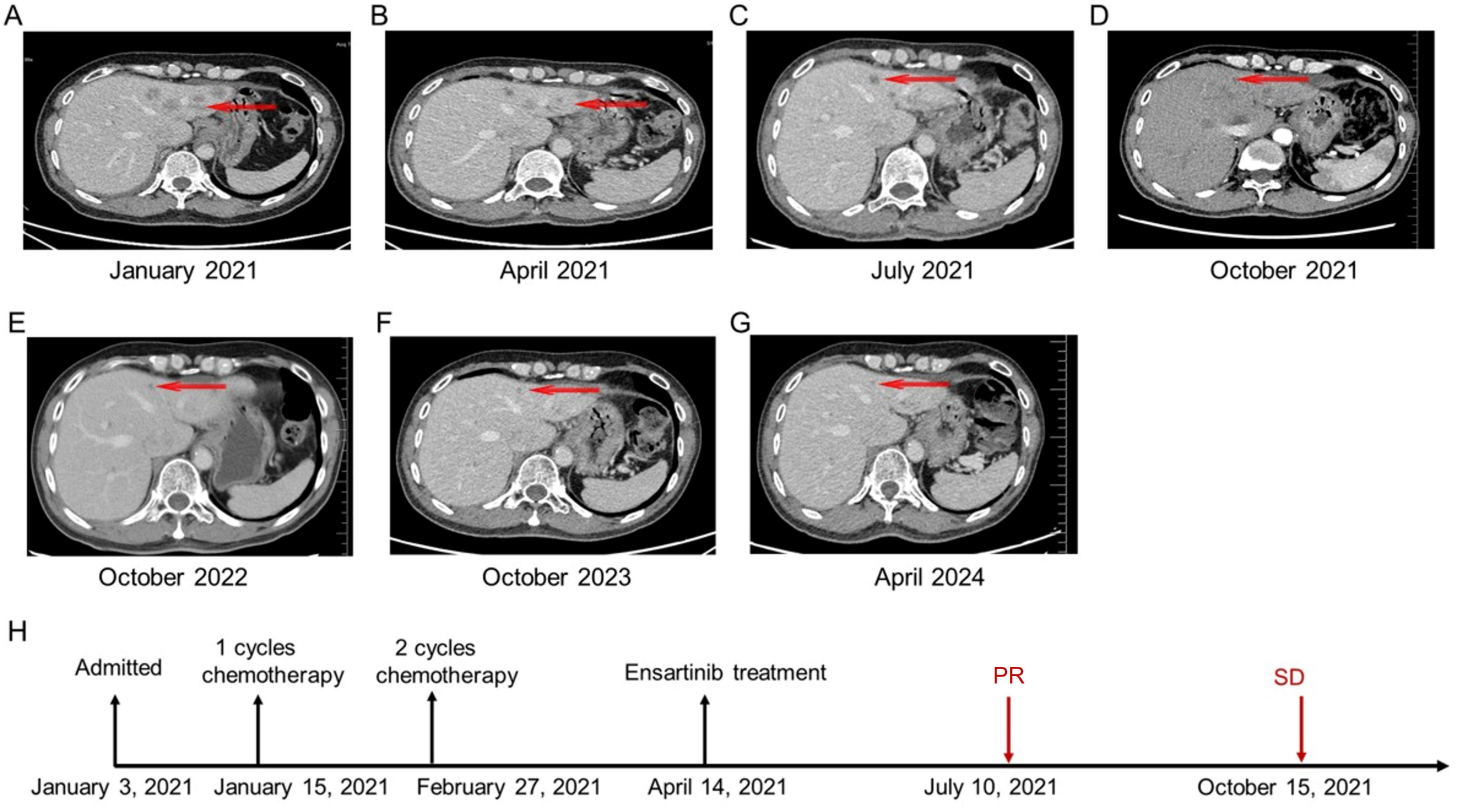
The patient’s chronological imaging follow-up results of liver lesions and treatment history. **(A)** CT of the abdomen before treatment: multiple liver metastases. **(B)** CT of the abdomen after 3 cycles of chemotherapy. **(C)** CT of the abdomen after 3 months of ensartinib treatment: PR of multiple liver lesions. **(D)** CT of the abdomen after 6 months of ensartinib treatment: SD in multiple liver lesions. **(E)** CT of the abdomen after 18 months of ensartinib treatment. **(F)** CT of the abdomen after 30 months of ensartinib treatment. **(G)** CT of the abdomen after 36 months of ensartinib treatment. **(H)** The patient’s treatment history. CT, computed tomography; PR, partial response; SD, stable disease.

From January 5 to January 13, 2021, he was preliminary diagnosed with advanced middle and lower esophageal adenocarcinoma with multiple metastatic tumors in both lungs and liver, and multiple lymph node metastases in the mediastinum, right hilum of the lung, bilateral common carotid artery, peritoneal cavity, and para-aorta by gastroscopy, gastroscopic ultrasound biopsy, thoracoabdominal computed tomography (CT) scan (**Figure 1**), and immunohistochemical (IHC) analysis by non-oncology departments. When the patient was referred to oncology specialists, prolonged pathology diagnosis that occurred before the referral delayed treatment. Given that imaging suggested the lesion could originate from either the esophagus or the lungs, and considering the patient’s PS score of 1, we promptly initiated chemotherapy to reduce the tumor burden on January 15, 2021(**Figure 2H**), with one cycle of albumin-bound paclitaxel (400 mg on day 1) and cisplatin (30 mg daily from day 3 to day 5). Due to the rarity of esophageal adenocarcinoma and complex imaging, our oncology specialists questioned the initial diagnosis. The next-generation sequencing (NGS) testing was performed on the seventh day of chemotherapy, revealing positive EML4-ALK rearrangement along with multiple point mutations in TP53, EGFR exon 20, and ERBB2 (which encodes human epidermal growth factor receptor 2 [HER2]) exon 20. Meanwhile, markers were added and re-immunohistochemical analysis was conducted, showing TTF-1 (+), CK7 (+), CK (+), Napsin A (+), Vimentin (−), CK5/6 (−), S-100 (−), P40 (−), CK20 (−), and Ki-67 (approximately 10%). PAS staining was also positive. Combined with the contrast-enhanced thoracoabdominal CT (**Figure 1**), the patient was ultimately diagnosed with lung adenocarcinoma (stage IVB, T4N3M1c) with ALK rearrangement and multiple gene mutations on February 23, 2021. Brain magnetic resonance imaging (MRI) revealed no metastatic lesions.

Based on diagnostic results and driver gene status, targeted therapy with ALK-TKIs was ultimately recommended. However, due to economic reasons, the patient declined this option and continued with chemotherapy. Given a reduction in tumor size following one cycle of chemotherapy and the worse prognosis associated with liver metastasis from lung cancer compared to other sites (13), from February 27 to April 14, 2021, the patient received two cycles of systemic chemotherapy, including albumin-bound paclitaxel (400 mg on day 1) and cisplatin (30 mg on days 1 and 2), followed by transcatheter arterial chemoembolization (TACE) with cisplatin (60 mg) and albumin.

**Figure 3:**
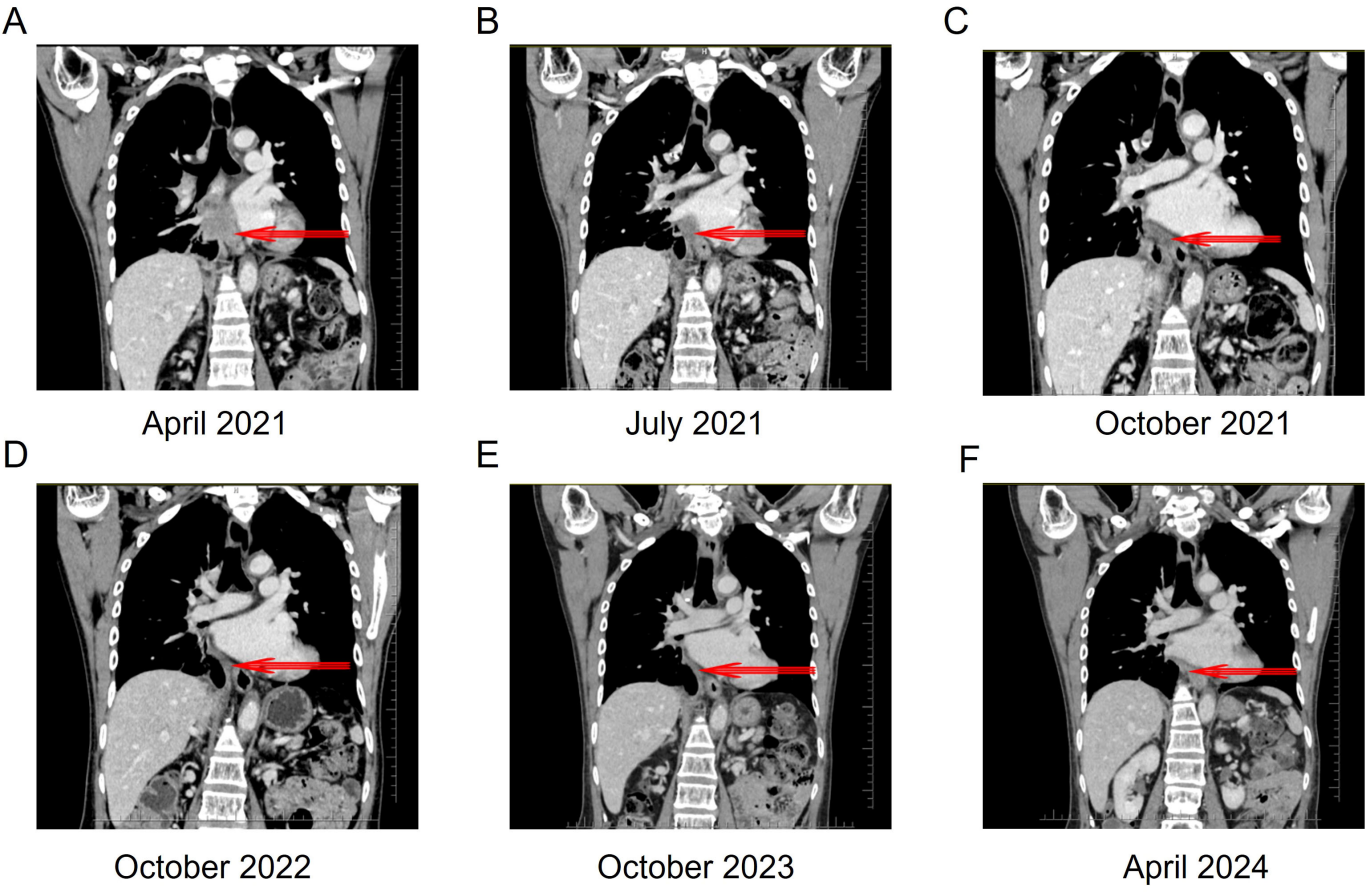
Thoracoabdominal computed tomography scan of the patient before and after ensartinib treatment. **(A)** Thoracoabdominal CT after 3 cycles of chemotherapy. **(B)** Thoracoabdominal CT after 3 months of ensartinib treatment: PR. **(C)** Thoracoabdominal CT after 6 months of ensartinib treatment: SD. **(D)** Thoracoabdominal CT after 18 months of ensartinib treatment. **(E)** Thoracoabdominal CT after 30 months of ensartinib treatment. **(F)** Thoracoabdominal CT after 36 months of ensartinib treatment. CT, computed tomography; PR, partial response; SD, stable disease.

**Figure 4:**
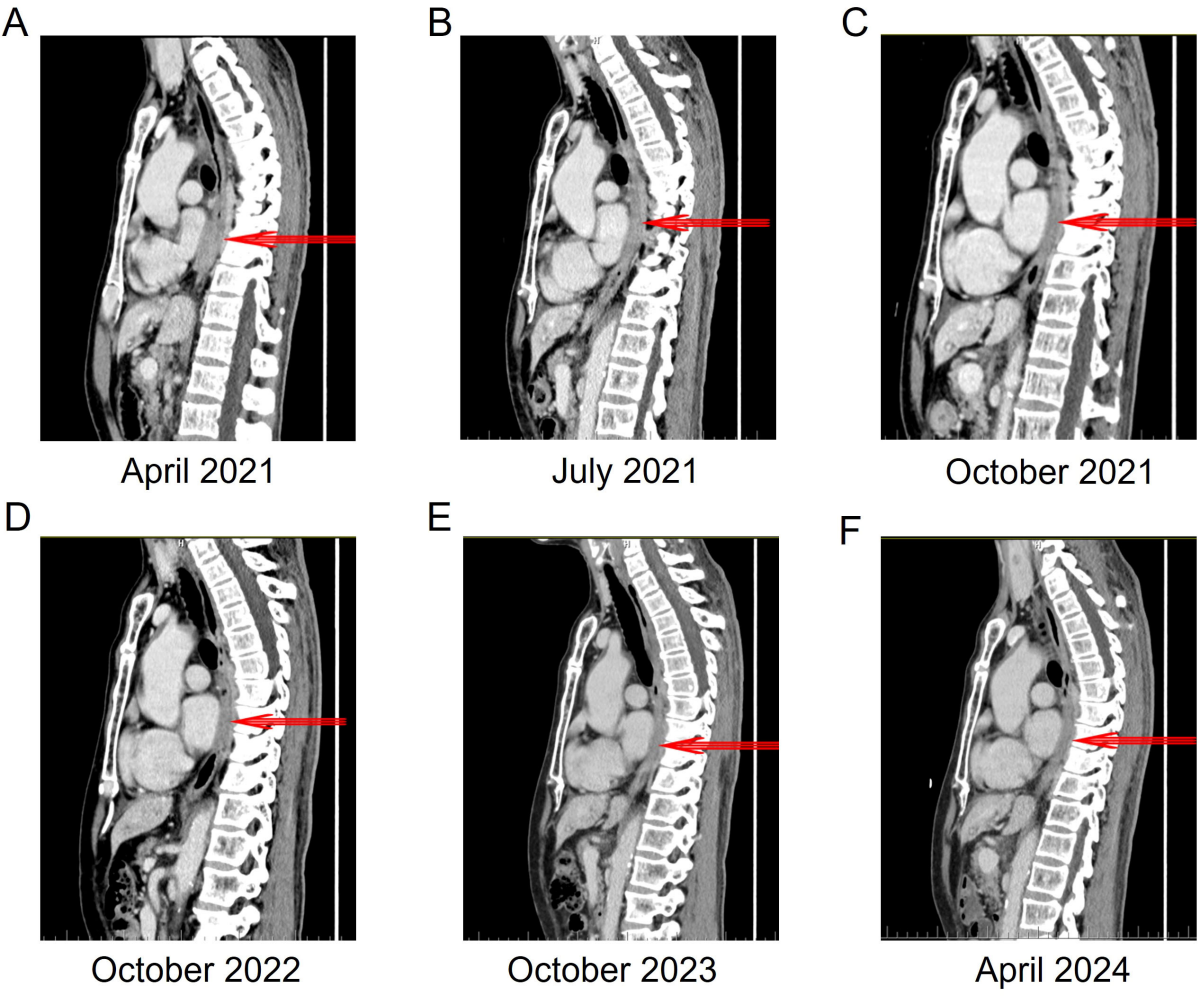
The changes in the local wall of the mid-to-lower thoracic esophagus within the mediastinum before and after ensartinib treatment. **(A)** 3 cycles of chemotherapy, the thickest part of the esophageal wall is 19 mm. **(B)** 3 months of ensartinib treatment, the thickest part of the esophageal wall is 13 mm. **(C)** 6 months of ensartinib treatment, the thickest part of the esophageal wall is 10 mm. **(D)** 18 months of ensartinib treatment, the thickest part of the esophageal wall is 10 mm. **(E)** 30 months of ensartinib treatment, the thickest part of the esophageal wall is 9 mm. **(F)** 36 months of ensartinib treatment, the thickest part of the esophageal wall is 8 mm.

After chemotherapy, the patient experienced mild nausea and a slight increase in alanine aminotransferase. Subsequently, the patient agreed to targeted therapy. Therefore, oral ensartinib hydrochloride (225 mg once daily) was initiated on April 14, 2021. After three months of ensartinib treatment (as of July 10, 2021), the patient’s multiple liver metastases notably decreased, resulting in a PR (**Figure 2C**, **3B**, **4B**). The patient’s condition remained in SD from October 15, 2021, to April 8, 2024, for approximately 30 months, with no brain metastases (**Figures 2**–**4**). The patient experienced a brief grade 1 rash during this period and is still on ensartinib treatment, without disease progression.

## 3 Discussion

In NSCLC, ALK gene rearrangements are usually detected in lung adenocarcinoma (14). To our knowledge, this is the first case of an advanced lung adenocarcinoma patient with EML4-ALK fusion and coexisting TP53, EGFR, and ERBB2 point mutations who was treated with ensartinib.

The detection of coexisting mutations of EGFR, HER2, TP53, and ALK rearrangement in this patient posed a challenge in choosing the appropriate TKI for treatment because there are no clinical guidelines specifying the order for using EGFR-TKIs and ALK-TKIs. It has been reported that a patient with both EGFR mutation and ALK rearrangement, who initially received treatment with an EGFR-TKI but experienced disease progression, subsequently achieved a PR with therapy using an ALK-TKI (7). A previous report also showed a decline in the overall response rate (ORR) among patients treated with EGFR-TKIs, whereas there was an increase in ORR for patients treated with ALK-TKIs (15). In addition, a patient with lung adenocarcinoma concomitant EGFR mutations and EML4-ALK fusion benefited from combination treatment with EGFR-TKIs and ALK-TKIs (16). This indicates that there is still controversy surrounding the treatment approaches for these cases. Therefore, for patients harboring multiple gene mutations, a personalized treatment strategy is necessary.

For patients with multiple gene alterations, prioritizing the primary driver mutation is the optimal strategy. In our case, the patient’s NGS testing revealed a higher variant frequency for EML4-ALK fusion (5.79%) compared to EGFR mutations (1.30%), indicating that ALK is the predominant oncogenic driver. The EML4-ALK fusion is the most common ALK rearrangement in NSCLC, and the most common variant 1 (v1; EML4 exon 13 and ALK exon 20) was detected in this case (17, 18). For advanced ALK+ NSCLC patients, ALK-TKIs are the first-line standard treatment (6). Additionally, an ALK+ NSCLC patient with multiple co-mutations responded well to ALK-TKIs (19). In our study, the patient harbored a rare type of EGFR exon 20 mutations (often in-frame insertions), which are usually unresponsive to EGFR TKIs approved for classical mutations (20). HER2 exon 20 mutants (often in-frame insertions) were also observed in this case, which similarly shows resistance to EGFR-TKIs (21, 22). EGFR exon 20 and HER2 exon 20 point mutations detected in the patient occur at relatively low frequencies, with limited reported data. Currently, there are no relevant guidelines or drug recommendations available.

Given the diagnostic results and driver gene status, ALK-TKI treatment was ultimately recommended. For this complex and refractory case, selecting an appropriate ALK inhibitor is crucial. In an eXalt3 study, ensartinib showed superior efficacy against both systemic and intracranial disease in ALK+ NSCLC patients compared with crizotinib (23). The most frequent toxic effects of ensartinib are rash and transaminitis, which are mostly low-grade, asymptomatic, and manageable (24). In conclusion, ensartinib, as a potent second-generation oral ALK-TKI with high activity against central nervous system metastases, has shown promising efficacy and acceptable safety for patients with ALK+ NSCLC (25). Therefore, ensartinib hydrochloride was received for targeted treatment after interrupting chemotherapy. The patient achieved PR after three months of ensartinib treatment and reached SD at six months, which was maintained for approximately 30 months. It has been proven that the patient achieved a durable drug response with ensartinib treatment. Aside from a mild rash, there were no other side effects. A recent case report demonstrated that a patient with an acquired EML4-ALK fusion, following resistance to osimertinib, received ensartinib and maintained a good quality of life over a 14-month follow-up period (26). Furthermore, a previously reported patient with metastatic ALK-rearranged lung cancer harboring an acquired ALK resistance mutation (ALK I1171N) showed a good response to ensartinib, with mild rash, and no further brain metastases after 3 months of follow-up (27). These results suggest that ensartinib is a potential treatment option for patients with ALK-rearranged lung adenocarcinoma.

Throughout the treatment process, we adhered to a shared decision-making approach with the patient and their family, ensuring that the treatment plan was professional and tailored to the patient’s actual situation. In response to the initial delays caused by non-oncology departments, we quickly initiated a chemotherapy regimen to reduce tumor burden and achieved the desired results. Furthermore, the differential diagnosis of diseases was successfully realized by accurate immunohistochemical and molecular pathological analysis, thereby earning the trust of the patient. Although follow-up genetic testing suggested that ALK inhibitors might be a better option, the patient chose to continue chemotherapy due to economic constraints. After three cycles, due to poor tolerance to chemotherapy, the patient transitioned to receive treatment of ensartinib. This therapy not only had mild side effects but also demonstrated significant efficacy, leading to high patient satisfaction, and the patient hopes to maintain SD in future standardized treatments.

This case highlights the importance of effective communication between doctors and patients and the value of personalized medicine, as well as serves as a valuable reference for similar cases in the future. Considering the good tolerability of current treatments, we may explore the combination of VEGF inhibitors like ramucirumab with ALK inhibitors in the future to further improve prognosis, provided that it remains tolerable for the patient (28). This strategy is primarily motivated by the observation that TP53 mutations, occurred at the highest frequency (30%) in the patient. TP53 mutations disrupt the tumor-suppressive function of the p53 protein and are associated with poorer objective response rates, shorter progression-free survival, and reduced overall survival when treated with ALK-TKIs or EGFR-TKIs (29, 30).

Overall, ensartinib showed potential in the treatment of EML4-ALK+ lung adenocarcinoma with multiple gene mutations. Nevertheless, whether the benefit of ensartinib can be achieved in larger patient cohorts remains unclear. Further investigation through clinical trials is warranted to assess the safety and efficacy of this targeted treatment, as well as its potential combination with other therapies.

## 4 Conclusions

In summary, we report the first case of an EML4-ALK+ lung adenocarcinoma patient with coexisting TP53, EGFR, and ERBB2 point mutations who achieved PR after three months of ensartinib treatment and reached SD at six months, which was maintained for approximately 30 months. This case provides a reference for managing EML4-ALK+ lung adenocarcinoma with multiple gene mutations.

## Data Availability

The original contributions presented in the study are included in the article/supplementary material. Further inquiries can be directed to the corresponding author.
